# Green Synthesis and Efficient Adsorption: Na-X Zeolite vs. C/Mn/SiO_2_ Composite for Heavy Metals Removal

**DOI:** 10.3390/ma17040954

**Published:** 2024-02-19

**Authors:** Magdalena Medykowska, Małgorzata Wiśniewska, Katarzyna Szewczuk-Karpisz, Mariia Galaburda, Olena Oranska, Rafał Panek

**Affiliations:** 1 Department of Radiochemistry and Environmental Chemistry, Institute of Chemical Sciences, Faculty of Chemistry, Maria Curie-Sklodowska University in Lublin, M. Curie-Sklodowska Sq. 3, 20-031 Lublin, Poland; 2 Institute of Agrophysics, Polish Academy of Sciences, Doświadczalna 4, 20-290 Lublin, Poland; k.szewczuk-karpisz@ipan.lublin.pl; 3 Chuiko Institute of Surface Chemistry, National Academy of Sciences of Ukraine, General Naumov Street 17, 03164 Kyiv, Ukraine; mariia.galaburda@gmail.com (M.G.); el.oranska@gmail.com (O.O.); 4 Department of Physicochemistry of Solid Surface, Institute of Chemical Sciences, Faculty of Chemistry, Maria Curie-Sklodowska University in Lublin, M. Curie-Sklodowska Sq. 3, 20-031 Lublin, Poland; 5 Department of Building Materials Engineering and Geoengineering, Faculty of Civil Engineering and Architecture, Lublin University of Technology, Nadbystrzycka 40, 20-618 Lublin, Poland; r.panek@pollub.pl

**Keywords:** synthetic zeolite, carbon-based composite, heavy metals removal, mixed adsorbate systems, electrical double layer, regeneration

## Abstract

The studies aimed to test the adsorption capacity of two silica-enriched porous materials, synthetic Na-X zeolite and Mn-containing carbon composite, towards Pb(II) and Zn(II) ions in single and mixed systems and in the presence of diclofenac (DCF) and (or) poly(acrylic acid) (PAA). The synthetic zeolite was characterized by a well-developed surface area of 728 m^2^/g and a pore diameter of 1.73 nm, while the carbon composite exhibited 268 m^2^/g and 7.37 nm, respectively. Na-X was found to be more efficient than the carbon composite (75–212 mg/g) in adsorbing heavy metal ions in both single and bimetallic systems (322–333 mg/g). In turn, the C/Mn/SiO_2_ composite was more effective in removing Pb(II) ions from the systems that simultaneously contained DCF or PAA (480 and 476 mg/g, respectively). The Na-X zeolite demonstrated the greatest stability in all the systems studied. The highest stability was observed in the DCF + Pb(II) mixture, in contrast to the carbon composites where the stability was much lower. To evaluate the possibility of regeneration of the solids, HCl proved to be the best desorbent for heavy metal ions (efficiency of 99%). In general, both adsorbents offer promising potential for solving environmental problems.

## 1. Introduction

Agricultural soils, surface water, and groundwater are confronted with pollution from various sources that jeopardize their suitability for plant growth. Pollution comes from farms, households, and hospitals whose wastewater contains various pollutants [1]. These pollutants include pharmaceutical degradation products, both biodegradable and non-biodegradable compounds, pathogenic bacteria, and metals (such as cadmium (Cd), copper (Cu), lead (Pb), zinc (Zn), and mercury (Hg)) [2,3]. However, heavy metal pollution of water and soil has gradually become a problem in recent years and can pose a serious threat to human health if left untreated [4]. Protecting soil health is crucial for sustainable agriculture and a healthier planet.

Nowadays, various methods such as chemical precipitation, ion exchange, reverse osmosis, and membrane separation can remove metal ions from wastewater [5]. However, in cases where the ion concentration is high (more than 100 mg/L), they are not efficient. It is important to note that the adsorption process has proven to be one of the most promising techniques for the treatment of waters containing toxic elements. The adsorption of heavy metal ions by zeolites and carbon-based composites has gained considerable credibility in recent years due to its low cost, ease of use, and high efficiency [6,7].

Zeolites are minerals composed of [AlO_4_] and [SiO_4_] tetrahedrons, forming a 3D lattice with channels and chambers arranged in supercells. This structure gives zeolites properties for sorption, catalysis, ion exchange, and molecular sieving [8]. They are widely used as adsorbents in environmental engineering, civil engineering, catalysis, and medicine [9,10]. Zeolites, used as additives in the composting of solid organic waste, contribute to better crop yields, water retention, and reduced loss of nutrients [11]. The used NaX zeolite was synthesized using waste after the production of zeolites from fly ash (carbon-combustion byproduct) in a hydrothermal conversion process, a method garnering interest due to its potential for waste reduction and environmental benefits [12].

It is worth mentioning that carbon–silica adsorbents also offer several advantages in the field of soil remediation and water treatment. In particular, carbon–silica adsorbents can efficiently remove hazardous substances from industrial wastewater and, thus, contribute to environmental protection [7]. The combination of carbon and silica provides a porous structure that increases adsorption efficiency. They are cheap, easy to use, and stable under different environmental conditions [13]. This stability is crucial for sustainable and effective remediation, especially in harsh or changeable environments. Moreover, they can be reused or recycled after use, and their durability ensures long-term performance [14]. They can also improve the soil quality and fertility [15].

In summary, zeolites and carbon-based adsorbents play a crucial role in addressing water/soil quality issues, making them valuable tools in environmental sustainability efforts.

This study demonstrates the potential of solids in the removal of heavy metal ions (Pb(II), Zn(II)), nonsteroidal anti-inflammatory drugs (diclofenac), and polymers (poly(acrylic acid)) from aqueous solutions. The selected adsorbates were selected due to their popularity and frequent occurrence in wastewater, as well as their toxic effect on plants and animals. Zinc, as a micronutrient, is essential for plant growth, but its excess in the soil can have various negative effects, such as disturbances in development and metabolism, ultimately leading to complete growth inhibition [16]. On the other hand, lead is a toxic element for plants and poses a threat to the life and health of animals and humans [17]. The pharmaceutical diclofenac, an over-the-counter medicine, and poly(acrylic acid), which is often used as a thickening agent in cosmetics and medicines, have high concentrations in wastewater [18]. In actual wastewater, this usually involves the coexistence of different metal ions. The adsorption of mixed metal ions under different conditions is more complex and can promote the interaction between the adsorbents or inhibit the effect of adsorption. Therefore, the coexistence of different states of biological adsorption of metal ions is very important. It is necessary to consider the adsorption mechanism of the mixed metal complex, the behavior of adsorption depending on the initial concentration of metal ions, the pH of the solution, and influencing factors such as the physical and chemical properties of the adsorbent.

The presented studies comprehensively describe the capabilities of the two silica-enriched adsorbents with respect to the removal of heavy metal ions from aqueous systems, both from simple and bimetal mixtures as well as from those containing organic substances. This is particularly important given the complexity of the natural samples. Due to the significant adsorption capacities of these carbon-based materials, they could potentially be used in the future as soil additives to reduce the bioavailability of heavy metals or pharmaceuticals to plants.

## 2. Materials and Methods

The study was carried out using the synthetic zeolite Na-X and the carbon–silica composite enriched with manganese C/Mn/SiO_2_. Zeolite was obtained by the hydrothermal reaction of an aqueous solution of sodium hydroxide and a byproduct of silica-rich waste solutions. C/Mn/SiO_2_, in turn, was synthesized in a two-step synthesis process. In the first step, phenol-formaldehyde polymer (*PFP, “Trading House Ukrainian Resins” Ltd., Kyiv, Ukraine*) was mechanochemically mixed with nanosilica (*A-300, pilot plant of the Institute of Surface Chemistry, Kalush, Ukraine*) and manganese(II) acetate tetrahydrate (*“pure” 98%, Sigma-Aldrich, St. Louis, MO, USA*) in a porcelain ball mill. The weight ratio of PFP–SiO_2_ was 1:1 and the metal content in the samples was 3 mmol/g SiO_2_. In the second stage, pyrolysis of the prepared mixture was carried out at 800 °C under an argon atmosphere [19]. The texture parameters of the solids were measured on an ASAP 2020 instrument (Micromeritics Instrument Corporation, Norcross, GA, USA) using low temperature nitrogen adsorption–desorption isotherms and are listed in Table 1. The XRD patterns were recorded using the DRON-UM1 diffractometer (Russia) and XPert Pro MPD (*Panalytical, Eindhoven, The Netherlands*). The SEM-EDS analyses were performed with a scanning electron microscope equipped with an energy-dispersive spectrometer (*Phenom ProX, Pik Instruments, Piaseczno, Poland*).

Two heavy metals, lead(II) and zinc(II), and two organic substances, polymer–poly(acrylic acid) (PAA), and the nonsteroidal anti-inflammatory drug diclofenac (DCF) (*Sigma-Aldrich, St. Louis, MO, USA*) were used as adsorbates. Both PAA and DCF have carboxyl groups of weak acidic properties and dissociate when the solution pH increases [20]. NaCl with a concentration of 0.001 mol/dm^3^ was used as a supporting electrolyte. In turn, HCl and NaOH with a concentration of 0.1 mol/dm^3^ were applied to establish the appropriate pH value of the examined suspensions as well as the desorbing agents.

Adsorption was conducted in the single and mixed adsorbate systems. The adsorbed amounts of Pb(II)/Zn(II) ions were examined at a temperature of 25 °C using a static method based on the change in adsorbate concentration before and after the adsorption process. The initial concentration for metals was 100 ppm, whereas for diclofenac and poly(acrylic acid), it was 50 ppm. The adsorption was carried out at pH 5 for 3 h in the case of the systems with Na-X and for 1 h in the systems containing C/Mn/SiO_2_. Emission atomic spectrometry with inductively coupled plasma (*Thermo Scientific iCAPTM 7200 ICP-OES analyzer, Waltham, MA, USA*) was applied to determine the concentration of heavy metals in the samples.

To determine the isoelectric point (iep) and zeta potential of the solid particles in the investigated suspensions, electrokinetic mobility measurements were conducted. The studies were carried out in the pH range of 3–10 using the Zetasizer Nano ZS instrument (*Malvern Instruments, Cambridge, UK*). Additionally, potentiometric titration was performed to determine the point of zero charge (pzc) and the surface charge density of the examined adsorbents. The titration setup included a thermostated Teflon container, glass and calomel electrodes (*Beckman Instruments, Brea, CA, USA*), a pH meter PHM 240 (*Radiometer, Copenhagen, Denmark*), laboratory stirrer, thermostat RE 204 (*Lauda, Lauda-Königshofen, Germany*), automatic burette Dosimat 765 (*Metrohm, Herisau, Switzerland*), and a computer equipped with the “titr_v3” software [21]. The concentrations of adsorbates were 10 ppm for Pb(II)/Zn(II) and 50 ppm for DCF/PAA.

The stability of the studied systems was investigated by a Cary 100 UV-VIS spectrophotometer (*Varian, Pal Alto, CA, USA*). The concentrations of adsorbates were 100 ppm for Pb(II)/Zn(II) and 50 ppm for DCF/PAA. The absorbance was measured at pH 5 as a function of time at a wavelength equal to 500 nm. A single measurement lasted 1 h and the individual results were recorded every 2 min.

## 3. Results

### 3.1. Characteristics of Na-X Zeolite and C/Mn/SiO_2_ Composite

**Figure 1:**
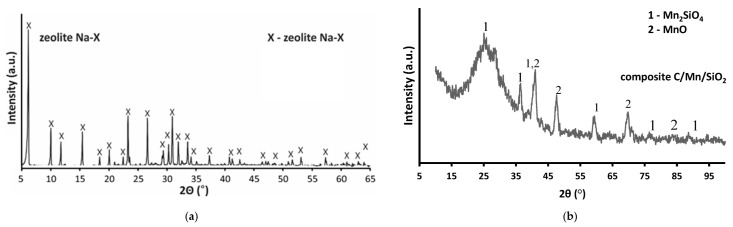
XRD patterns for Na-X zeolite (**a**) and C/Mn/SiO_2_ composite (**b**).

The XRD patterns of the Na-X zeolite and the C/Mn/SiO_2_ composite are shown in Figure 1. The Na-X zeolite is represented by a series of characteristic diffraction peaks in the 2θ range of 5–50°. No other reflexes were observed [22]. Additionally, within the angular range of 15–35 degrees 2θ, the diffractograms did not exhibit an increase in background line, a distinctive characteristic often observed in zeolites derived from fly ash and attributed to the existence of an amorphous phase. The zeolites exhibited high purity monomineral zeolitic phases, which closely matched those of commercially available Na-X zeolites. For the C/Mn/SiO_2_ composite, the main phases were Mn_2_SiO_4_ (PDF card 74–716) and MnO (PDF card 75–625). A halo of amorphous silica was observed in the pattern at 2θ = 25°, while the formation of graphite structures was not detected.

**Figure 2:**
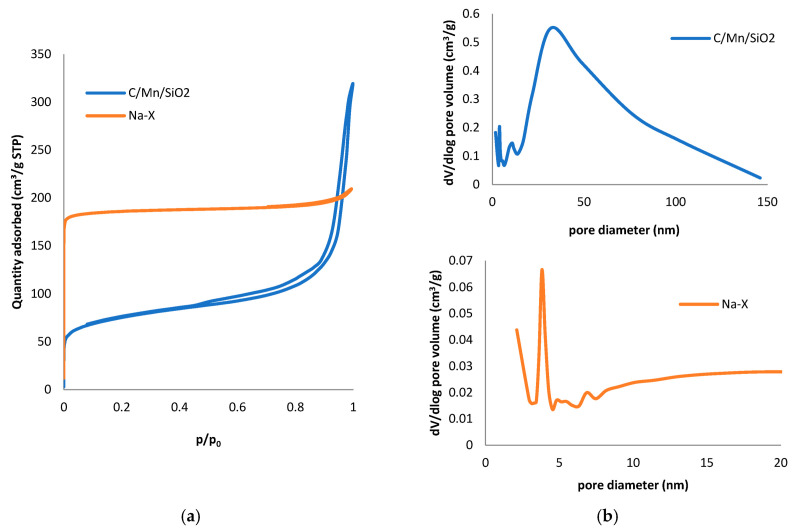
Nitrogen adsorption/desorption isotherms (**a**) and pore size distribution (**b**) for Na-X zeolites and C/Mn/SiO_2_ composite.

The nitrogen adsorption/desorption isotherms obtained for the adsorbents investigated and the pore size distributions are shown in Figure 2. The calculated texture parameters, including the specific BET surface area, micropore area and micropore volume, total pore volume, and average pore radius, are summarized in Table 1. The sorption and desorption isotherms for both composites are a mixture of I and IV types according to the IUPAC classification, with high N_2_ uptake at low relative pressure (*p*/*p*_0_ < 0.1) and with a H4 hysteresis loop characteristic of systems with micropores and mesopores [23]. The specific surface area was determined to be 268 m^2^/g for C/Mn/SiO_2_. The blue curve in Figure 2b shows a broad pore size distribution in the carbon sample with some main peaks at R = 0.5, 1.6–1.8, and 18 nm and a prominent peak at ~30 nm, demonstrating the presence of micro- and mesopores as well as a complex texture of the selected solid. The Na-X zeolite was characterized by a much more developed surface area than the carbon-based composite with a specific surface area of 728 m^2^/g. Significant nitrogen absorption at low relative pressure is observed, followed by a flat region in the isotherm, indicating microporosity in these materials. At the same time, a weak hysteresis loop is also observed, which is due to the presence of a small amount of mesopores. The micropore area in Na-X was more than 85%, while it was estimated to be ~43% in the carbon composite.

**Figure 3:**
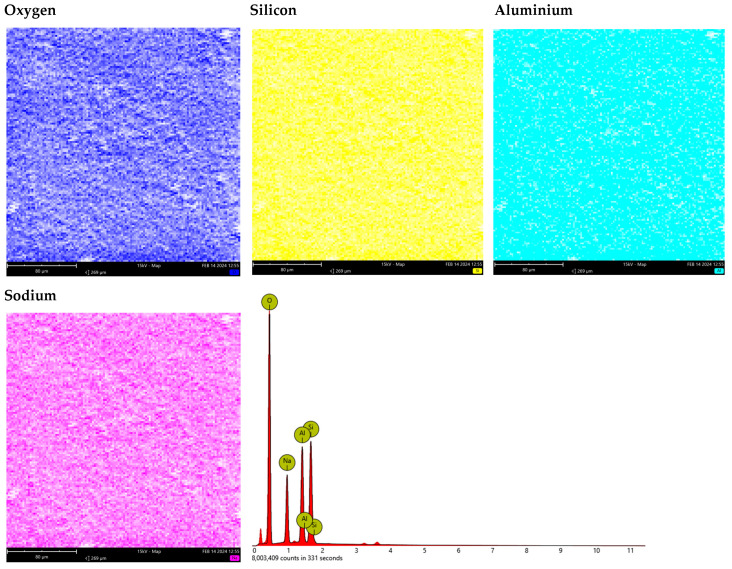
Elemental composition of Na-X zeolite determined by SEM-EDS.

**Figure 4:**
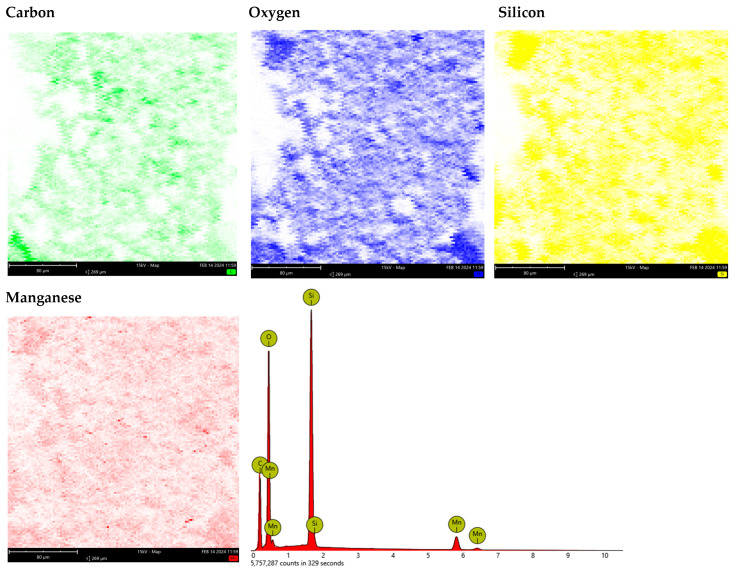
Elemental composition of C/Mn/SiO_2_ determined by SEM-EDS.

Figure 3 and Figure 4 present the results of the SEM-EDS analyses for Na-X and C/Mn/SiO_2_. As can be seen, Na-X was composed of 64.87% oxygen (O), 13.06% silicon (Si), 11.45% aluminum (Al), and 10.63% sodium (Na). In turn, C/Mn/SiO_2_ was composed of 44.95% carbon (C), 42.11% O, 10.73% Si, and 2.21% manganese (Mn).

### 3.2. Comparison of Adsorption/Desorption of Heavy Metal Ions on the Na-X Zeolite and the C/Mn/SiO_2_ Composite in the Single and Mixed Adsorbate Systems

The adsorption isotherms and kinetics of heavy metals on Na-X and C/Mn/SiO_2_ were presented elsewhere [19]. In turn, the results of adsorption data modeling using Langmuir and Freundlich models are summarized in Table 2.

For Na-X, experimental isotherms were better described by the Langmuir model (R^2^ = 0.966–0.997). This phenomenon is typical of homogenous materials. During ion adsorption on their surface, one ion interacts with one active site, and all adsorption sites are energetically identical. On the other hand, the ion adsorption on C/Mn/SiO_2_ was best fitted to the Freundlich model (R^2^ = 0.976–0.994). This model describes the adsorption of metal ions on heterogenous materials, whose active sites are not energetically equivalent [24]. For both investigated materials, the adsorption kinetics of Pb(II)/Zn(II) were best described by a pseudo-II-order equation, which indicated the chemical character of this process (chemisorption). Between the adsorbent and adsorbate, chemical bonds through the exchange or sharing of electrons were created [25]. Metallic elements (Mn) in the composite structure are additional sites for metal cations to adsorb. During Mn incorporation, M-OH groups were introduced to the solid surface. They interacted with metal ions and formed multiple complexes (e.g., Pb-O-M) based on various mechanisms [26].

**Figure 5:**
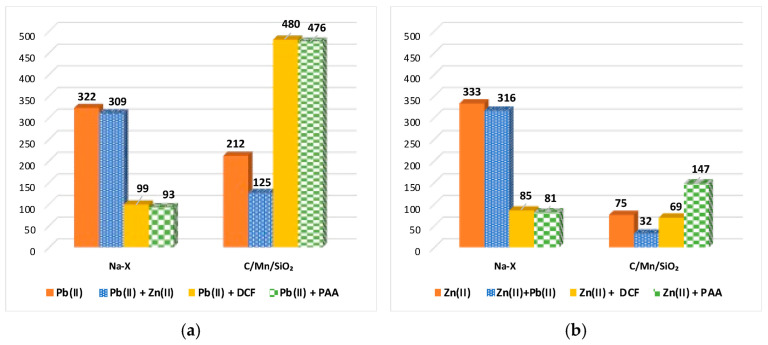
Adsorbed amounts of Pb(II) (**a**) and Zn(II) (**b**) ions in the single and mixed systems with Zn(II)/Pb(II)/DCF/PAA on the surface of Na-X and C/Mn/SiO_2_.

Figure 5a,b present the adsorbed amounts of Pb(II)/Zn(II) ions on the surface of Na-X and C/Mn/SiO_2_ materials measured in the single and mixed systems. In the mixed systems, in addition to the ions of the selected heavy metal, ions of the second metal, DCF or PAA were also present. It was observed that zeolite had better efficiency in the adsorption of heavy metal ions in the single systems, with both lead(II) and zinc(II) adsorbed more readily on the Na-X surface than the C/Mn/SiO_2_ one. This can be related to the fact that the zeolite has an almost three times larger specific surface area than the carbon–silica adsorbent (Table 1). In the single systems, the level of heavy metal adsorption on Na-X remained practically identical (in the range 322–333 mg/g), whereas on C/Mn/SiO_2_, it differed more clearly (in the range 75–212 mg/g). On the composite, Pb(II) cations were adsorbed more effectively than Zn(II) ones. This phenomenon can be due to differences in the porous structure of these two adsorbents, which favored lead(II) ions adsorption. In the mixed adsorbate systems containing two different heavy metals, a slight decrease in adsorption was observed for Na-X and a much larger decrease for C/Mn/SiO_2_. Once again, the significant specific surface area of the zeolite was responsible for this tendency. The decrease in heavy metal ions adsorption in the mixed systems was caused by competition for active sites of the applied materials [27]. In addition, both adsorbents under the experimental conditions, i.e., at pH 5, have a positively charged surface. The obtained high adsorption levels of both metal cations mean that the selected adsorbents are very effective, even under unfavorable electrostatic conditions occurring between the solid surface and the inorganic ions. This behavior revealed the occurrence of specific interactions in the adsorption system, such as chemical bonds, both hydrogen and polar covalent ones.

In contrast, in the mixed systems containing zeolite and organic substances, the decrease in lead(II) and zinc(II) ions adsorption was noticeable. Both organic substances of anionic character, DCF and PAA, had a higher electrostatic affinity for the solid surface than heavy metal ions. In addition, the adsorbates (metal cations and organic anions) can form complexes in the solution, both intermolecular and intramolecular, which can result in the decrease in heavy metal ion adsorption. However, in the mixed systems containing heavy metal–DCF, heavy metal–PAA, and the composite, an increase in heavy metal ion adsorption was observed. In the case of lead(II) ions, there was a significant increase in ion adsorption due to the addition of the drug or polymer. For zinc(II) ions, the presence of DCF slightly decreased metal adsorption, whereas the addition of PAA had the opposite effect. This was probably due to the previously mentioned differences in adsorbent structure, which can favor Pb(II) ions. With practically a four times larger mean pore diameter, the composite seemed to be able to adsorb the formed complexes of metal ions–organic substance, which significantly increased the adsorption level of lead(II) cations. Detailed comparative information can be found in Table 3 below.

**Figure 6:**
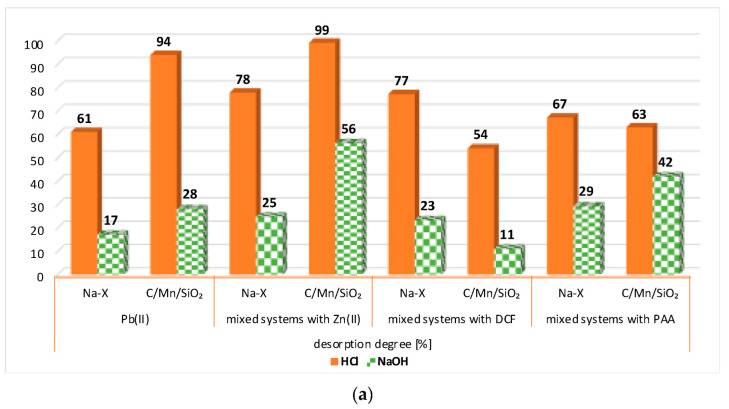

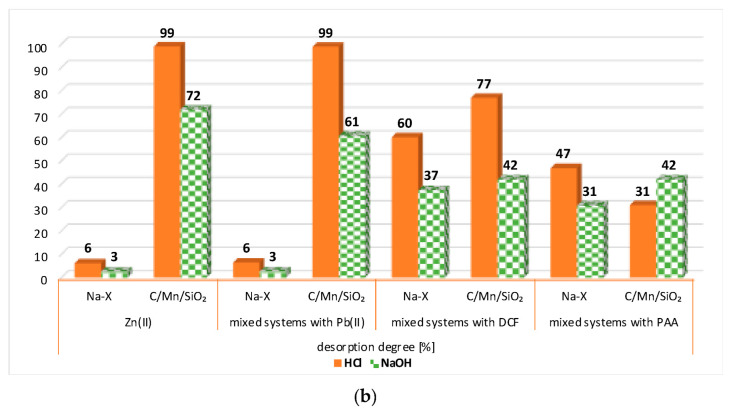
Desorption degree of Pb(II) (**a**) and Zn(II) (**b**) ions in the single and mixed systems with Pb(II)/Zn(II)/DCF/PAA from the surface of Na-X and C/Mn/SiO_2_.

Figure 6a,b present the degree of desorption of Pb(II)/Zn(II) ions from the surface of Na-X and C/Mn/SiO_2_ in the single and mixed systems of Pb(II)/Zn(II) and DCF/PAA. For practically all investigated systems, it can be concluded that hydrochloric acid turned out to be the best desorbing agent for heavy metal ions. Generally, the lead(II) ions were desorbed by HCl in larger amounts from the solid surface (even in 99%).

The high percentage of desorption confirmed the high regenerative abilities of the applied adsorbent. This is highly important from the point of view of their reuse in subsequent adsorption cycles.

### 3.3. Comparative Electrokinetic Studies

**Figure 7:**
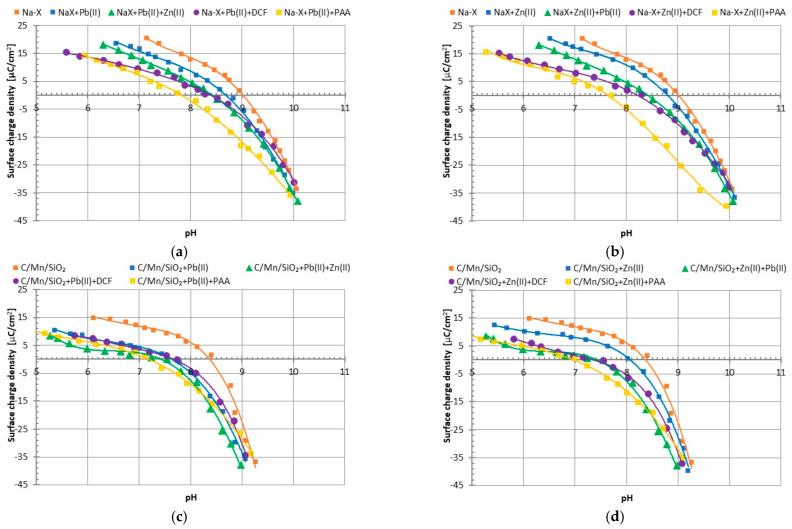
Surface charge density of Na-X (**a**,**b**) and C/Mn/SiO_2_ (**c**,**d**) particles in the presence of Pb(II) (**a**,**c**) and Zn(II) (**b**,**d**) ions in the single and mixed systems with Pb(II)/Zn(II)/DCF/PAA.

The surface charge density (σ_0_) is a very helpful parameter for obtaining information about the sign and density of the surface charge assumed by the selected solid in aqueous suspension. At the pH values above the point of zero charge (pH_pzc_), the solid takes on the positive sign of charge, whereas below pH_pzc_, its sign of charge is negative. The point of zero charge (pzc) is the pH value, at which the concentrations of positively and negatively charged surface groups are the same. As can be seen in Figure 7a–d, under experimental conditions (i.e., pH 5) both solids were characterized by a positive surface, which was already mentioned before. The pH_pzc_ value for Na-X was about 9.0, whereas that for C/Mn/SiO_2_ was about 8.4. The decrease in surface charge density due to the addition of heavy metal ions was observed for all the systems studied. In highly complex systems, the decrease in this parameter was influenced by a few effects accompanying the adsorption of different types of compounds (differing significantly in molecule size and ionic character). In the case of the adsorption of small divalent metal cations, the σ_0_ decrease was related to the formation of an additional number of negatively charged groups on the adsorbent surface (due to the electrostatic interactions of its surface groups with metal ions). In contrast, for the adsorption of large anionic molecules, such as drug or long-chain polymer ones, the decrease in surface charge density was caused by the presence of negatively charged groups belonging to PAA or DCF in the by-surface layer of the solution. These were not directly bound to the surface of the examined materials, indicating the presence of other interactions in addition to electrostatic forces, e.g., hydrophobic forces or hydrogen bonds. Such a course of the experimental curves was certainly influenced by the phenomenon of complex formation between the adsorbates of different ionic characters or their competition for active sites on the surfaces of applied adsorbents [35]. In the case of adsorption layers with very complex structures, the resultant surface charge was determined by both ions or polymer train segments directly connected to the active sites on the solid surface, as well as charged chemical entities (metal ions, polymeric functional groups belonging to loop, and tails segments) located in the near-surface layer in the rigid Stern layer.

**Figure 8:**
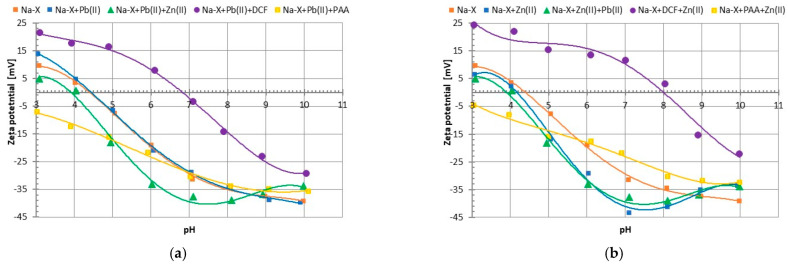

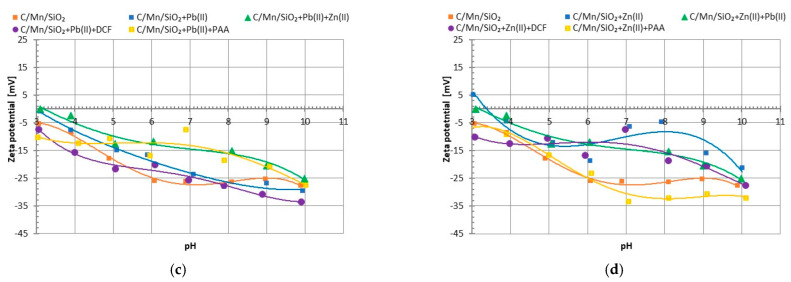
Zeta potential of Na-X (**a**,**b**) and C/Mn/SiO_2_ (**c**,**d**) particles in the presence of Pb(II) (**a**,**c**) and Zn(II) (**b**,**d**) ions in the single and mixed systems with Pb(II)/Zn(II)/DCF/PAA.

Another important parameter characterizing colloidal suspensions is the zeta potential (ζ), i.e., the potential of the slipping plane separating the stiff part of the electrical double layer directly attached to the solid surface from its movable diffusion part. Figure 8a–d show the dependencies of this parameter on the solution pH value. In the case of Na-X, the pH_iep_ is 4.4, and for C/Mn/SiO_2_, it is out of the examined pH range (below pH 3). The solid isoelectric point (iep) characterizes such pH conditions (pH_iep_), at which the concentration of positive and negative charges (inorganic ions, dissociated functional groups belonging to the adsorbed organic molecules) located in the slipping plane area is the same.

In most systems containing diclofenac (especially with Na-X), the zeta-potential values were increased in comparison to the analogous systems without adsorbates. In turn, in the case of the composite, for the systems with Pb(II) ions, the greatest decrease was visible when DCF was added, and for systems with zinc(II), when PAA was added. In the examined suspensions, many effects affected the zeta-potential values. The addition of PAA usually reduced this parameter due to the presence of negatively charged carboxyl groups of the polymeric chain (belonging to segments in loop and tail structures) in the slipping plane area, but, also, this phenomenon can be dictated by the displacement of the slipping plane from the solid particle’s surface by the adsorbed macromolecules forming thick adsorption layers. On the other hand, the increase of zeta potential due to DCF addition was caused by the transfer of supporting electrolyte counterions from the surface layer to the diffusive part of the electrical double layer. The addition of small heavy metal cations usually reduced the zeta potential by their adsorption, causing the supporting electrolyte Na^+^ ions to be pushed out from the active surface sites towards the slipping plane area. In such complex systems, changes in the zeta potential were also influenced by the formation of complexes between adsorbates (organic substance–metal), but additionally by the competitive adsorption of small metal cations, which affected the ionic composition of the diffusion part of the electrical double layer [36].

### 3.4. Comparison of Stability of the Zeolite and Composite Suspensions

**Figure 9:**
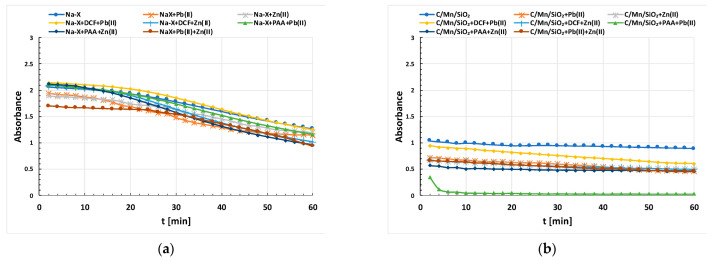
Stability of Na-X (**a**) and C/Mn/SiO_2_ (**b**) suspensions in the presence of Pb(II) and Zn(II) ions in the single and mixed systems with Pb(II)/Zn(II)/DCF/PAA.

The time dependency of absorbance of the examined suspensions after the addition of adsorbates of different natures is shown in Figure 9a,b. Among the systems studied, the more stable suspensions at pH 5 were those formed by the Na-X particles. Regardless of the addition of adsorbates, the absorbance values characterizing these systems were higher than those obtained for systems containing the composite. In the case of Na-X suspensions, the stability of all systems decreased in fairly similar dynamics over time. The systems containing two heavy metal ions simultaneously, as well as the mixed systems with Zn(II) + DCF and Zn(II) + PAA adsorbates, were characterized by the relatively lowest stability. The suspension containing zeolite with DCF + Pb(II) exhibited the greatest stability, and the same behavior was observed for the system containing the composite itself. Among the systems containing C/Mn/SiO_2_, the least stable suspension was the one containing Pb(II) + PAA. Excluding this system, in most of the suspensions formed by C/Mn/SiO_2_ particles, the decrease in absorbance over time was relatively small.

The stability of complex samples was determined by different effects coming from each adsorbate. For example, the addition of the polymer can cause a flocculation phenomenon and the formation of easily sedimenting aggregates (destabilization of suspension occurs). This is related to the formation of polymer bridges, i.e., through the adsorption of the long polymeric chain on at least two solid particles surfaces. In addition, both PAA and DCF molecules with anionic character also have the effect of deterioration of the system stability on the way of the solid surface charge neutralization by the oppositely charged adsorbate. In contrast, coagulation and the associated decrease in stability often take place after the addition of heavy metal ions. In the mixed systems, the formation of complexes between the adsorbates is certainly also an important factor. Hence, in the mixed adsorption layers composed of metal–organic substance complexes, steric and electrosteric mechanisms are primarily accountable for the suspension stability [37]. These steric/electrosteric forces lead to the rise in the repulsion of solid particles covered with mixed adsorption layers and the improvement of stability conditions in the examined suspensions.

## 4. Conclusions

Through the analysis of the results obtained, the following conclusions were drawn. Zeolite shows better efficiency in the adsorption of heavy metal ions, either from the systems containing one metal or both metals simultaneously. Na-X has a much larger specific surface area, but C/Mn/SiO_2_ is characterized by a larger mean pore diameter. The decrease in adsorption in the systems with both heavy metals is related to their competition for active sites on the surface of the adsorbents. In contrast, in the mixed systems with organic substances, the zeolite performed a worse adsorption capacity, whereas heavy metal adsorption by C/Mn/SiO_2_ in the mixed systems with DCF and PAA was significantly higher than those in the single systems. Hydrochloric acid proved to be the best desorbing agent, and desorption occurred at a fairly high level, which may indicate the high regeneration potential of these materials. Electrokinetic studies showed that under the adsorption conditions, i.e., pH 5, both materials had a positively charged surface, which may suggest that the drug and polymer with anionic characters adsorption were favored electrostatically. As a result of the adsorbates’ addition in the single and mixed systems, there was a reduction in the surface charge density in all suspensions, which was the sum of the effects of the adsorption of each adsorbate. In the case of the zeta potential, the cumulative effects for the different systems varied and so, for the system with Na-X and Pb(II) + DCF, the potential increased, but for the same system with C/Mn/SiO_2_, it decreased. The stability of the suspensions formed by the zeolite particles was at a higher level than that formed by the composite particles. Also, the stability of the adsorbent particles decreased in most cases due to the addition of adsorbates, suggesting a high potential in terms of their separation from the solution after the adsorption process. Both investigated materials, as representatives of the two groups of adsorbates, have great potential and may prove successful in removing heavy metals from complex systems such as environmental samples.

## Figures and Tables

**Table 1:** Physicochemical characteristics of the Na-X zeolite and the C/Mn/SiO_2_ composite.

Sample	BET Surface Area [m^2^/g]	Micropore Area [m^2^/g]	Pore Volume [cm^3^/g]	Micropore Volume [cm^3^/g]	Mean Pore Diameter [nm]
Na-X	728	624	0.31	0.27	1.73
C/Mn/SiO_2_	268	114	0.49	0.05	7.37

**Table 2:** Isotherm parameters of Pb(II) and Zn(II) adsorption on the Na-X and C/Mn/SiO_2_ at pH 5.

		Langmuir Model			Freundlich Model		
System		q_m_ [mg/g]	K_L_ [dm^3^/mg]	R^2^	n	K_F_ [mg/g (mg/dm^3^)^−1/nF^]	R^2^
Pb(II)	Na-X	676.594	0.227	0.966	1.625	0.0005	0.794
	C/Mn/SiO_2_	372.492	0.025	0.876	3.950	0.004	0.994
Zn(II)	Na-X	693.289	4.878	0.997	2.148	0.0002	0.943
	C/Mn/SiO_2_	149.484	0.013	0.979	1.611	0.012	0.976

**Table 3:** Adsorption capacity of adsorbents for Pb(II) and Zn(II) with and without organic additives.

Material	Q_m_ [mg/g]			Organic Additives	Reference
	Pb(II)	Zn(II)	Pb(II)/Zn(II)	-	
Zeolitic adsorbent ZCET40	322.58	69.93	238.10/69.93	-	[28]
Zeolitic adsorbent ZDs40	277.78	61.35	185.1/6.35	-	[28]
β-Cyclodextrin/ZrO_2_	274.4	-	-	Bisphenol A	[29]
Fe-NPs	100	16.74	-	Rifampicin	[30]
Montmorillonite with alkyl-ammonium and complexant		16.74	-	p-nitrophenol	[31]
EDTA and chitosan bifunctionalized magnetic bamboo biochar	63.2 (Cd(II))	50.8	-	methyl orange (305.4 mg/g)	[32]
Biochar-based sorbents	19.1	19.2	-	-	[33]
Plant-based biochars	0.344	1.081	-	-	[34]

## Data Availability

Data are contained within the article.
